# A Low-Cost Assistive Robot for Children with Neurodevelopmental Disorders to Aid in Daily Living Activities

**DOI:** 10.3390/ijerph18083974

**Published:** 2021-04-09

**Authors:** Roberto J. López-Sastre, Marcos Baptista-Ríos, Francisco Javier Acevedo-Rodríguez, Soraya Pacheco-da-Costa, Saturnino Maldonado-Bascón, Sergio Lafuente-Arroyo

**Affiliations:** 1 GRAM, Department of Signal Theory and Communications, University of Alcalá, 28805 Alcalá de Henares, Spain; javier.acevedo@uah.es (F.J.A.-R.); saturnino.maldonado@uah.es (S.M.-B.); sergio.lafuente@uah.es (S.L.-A.); 2 Multimodal Information Group, Gradiant, 36310 Vigo, Spain; mbaptista@gradiant.org; 3 FINEMEV, Department of Nursing and Physiotherapy, University of Alcalá, 28805 Alcalá de Henares, Spain; soraya.pacheco@uah.es

**Keywords:** artificial intelligence, assistive technology, robotics, action detection

## Abstract

In this paper, we present a new low-cost robotic platform that has been explicitly developed to increase children with neurodevelopmental disorders’ involvement in the environment during everyday living activities. In order to support the children and youth with both the sequencing and learning of everyday living tasks, our robotic platform incorporates a sophisticated online action detection module that is capable of monitoring the acts performed by users. We explain all the technical details that allow many applications to be introduced to support individuals with functional diversity. We present this work as a proof of concept, which will enable an assessment of the impact that the developed technology may have on the collective of children and youth with neurodevelopmental disorders in the near future.

## 1. Introduction

According to the Spanish Ministry of Health and Social Affairs [1,2], up to 74% of the Spanish population with disabilities over 6 years old has difficulties performing activities of daily living (ADLs). Mobility, domestic life and self-care limitations are the main groups of disabilities. This situation has an important negative impact on people’s participation in different environments.

The International Classification of Functioning, Disability and Health (ICF) [3] provides a common language to describe human functioning. ICF describes disabilities as deficits at the level of body structures and functions, limitations in individual functional activity and restrictions in social participation, in the context of environmental and personal factors that can interact with each other. Environmental factors include assistive technology (AT), which is any product, instrument, strategy, service or practice used by people with disabilities to prevent, compensate, relieve or neutralize an impairment, disability or handicap. AT must improve the individual’s autonomy and quality of life (QoL) [4].

The ICF version for children and youth (ICF-CY) [5] is often used as a biopsychosocial model to guide selection measures, treatment goals and outcomes for children and youth with neurodevelopmental disorders (NDDs). This community presents physical, sensory, learning and/or communication impairments. Within this context, AT may act as a facilitator in meaningful activities in which people with NDDs are able to participate [6]. Therefore, assistive robotic devices (ARDs) can be used for improving age-appropriate function, increasing independence and encouraging learning. The reason is clear: ARDs enhance the ability of people with NDDs to be fully engaged in activities at home, at school, in healthcare settings and within their communities [7].

One of the main purposes for management and interventions of children and youth with NDDs is to increase functionality and autonomy in ADLs, in order to improve their QoL and participation within all spheres of life. Participation in home, school, community and leisure activities is of the utmost importance to those with NDDs and their families. The intensity of participation is usually influenced by multiple factors, and people with NDDs with significant mobility impairments, who cannot move independently, are at risk of additional secondary mobility-related, socialization and learning limitations. Therefore, an ARD that could be used as a companion may facilitate independent mobility.

On the other hand, learning daily living tasks is also difficult for people with NDD. E.g., we can think about brushing teeth, which is a quite simple daily routine that has a sequence of steps that should be performed in a certain way/order. An ARD that could be able to recognize and monitor actions, which are captured in real-time with a camera embedded in a robotic platform, could be quite useful for activity learning. Fortunately, action recognition in videos is a growing research topic in computer vision and artificial intelligence [8]. The idea is simple: video sequences captured by the camera of an ARD are processed with an action detection module, which has been previously trained to recognize a set of action categories. The robot then should use this information to deploy a set of applications to improve the participation in the environment through ADLs for users with NDDs. This is actually the main objective of our research.

Overall, the main contributions of our work are the following:

1. We introduce a novel, low-cost assistive robot to attend to kids and youth with NDDs. Note that all of the mechanical design of the platform is new; therefore, we do not need to use any expensive commercial solution. Moreover, this aspect allows us to perform any (mechanical) adaptation that the final user might need. This is a fundamental feature for an ARD, because the final design must ensure the user’s satisfaction and usefulness, to avoid difficulties and disappointments during its use [9].
2. Our robotic platform integrates an online action detection approach for monitoring the development of ADLs of the users.
3. We propose two applications specially designed to assist children with NDDs. The first one helps the kids to develop a correct sequencing of ADLs. The second application focuses on aiding the users to practice and learn a specific action. Both solutions assist the users to improve their independence in ADLs.
4. We offer a detailed experimental validation of the online action detection module, using a well-known action recognition dataset; we are able to report an accuracy of 72.4% for 101 different action categories.
5. We consider this work as a proof of concept, allowing for a potential evaluation of the impact that the developed technology will have on a group of children and youth with NDDs.

The rest of the paper is organized as follows. In Section 2 we provide a review about state-of-the-art ARDs and about the role of action detection solutions in this field. Section 3 describes our novel low-cost robotic platform. We provide details concerning the hardware and software architectures implemented in Section 3.1 and Section 3.2, respectively. Section 3.3 is used to introduce the proposed online action detection solution. In Section 4 we describe the implemented applications for monitoring ADLs of users with NDDs. We provide an experimental validation of the action recognition software (Section 4.1), and qualitative results (Section 4.2). Finally, conclusions are presented in Section 5.

## 2. Related Work

Traditionally, ARDs are used as learning robots for activities at different environments: (a) as part of school curricula; (b) for therapy interventions; and (c) for other activities that take place at home and in communities. In a recent systematic review about educational robotics for children with NDDs [10], the authors reported that the most common learning robots are Bee-Bot, KIBO, Lego MINDSTORMS and NAO NextG. The study concluded that learning robots improve performance in learning objectives, and the children’s engagement in learning activities and communication/interactions with peers. Moreover, teachers and families reported the experience as positive.

Socially assistive robotics are emerging in the pediatric field [11]. They involve physically and socially assistive robots (PSAR) that help through advanced interactions, such as: companionship, playing, tutoring, physical therapy and daily life assistance. Dawe et al. [12] concluded in their study that the main roles of social robots are: acting as a companion, providing entertainment and distraction, increasing motivation and joy, expressing empathy, being a buddy for playing/learning and coaching for information provision and exercise demonstration. The most popular PSAR for children with NDD are IROMEC, MOnarCH, NAO, Puffy, Robot-avatars, SPELTRA and Teo. Companion robots, such as IROMEC, Paro and Teo, play the role of a social mediator. The interactions with the robot also promote human–human interaction, develop social skills and improve QoL in children with NDDs [13].

ARDs can be classified into three categories:

1. Fixed home adaptations.
2. Wheelchair solutions.
3. Mobile platforms.

Fixed home adaptations focus on building smart environments in the home of the user, such as: micro-rooms [14,15], smart bathrooms [16] and smart kitchens [17]. These examples are inspiring. However, they are extremely specialized. They lack the capacity of being easily integrated into a regular home or home care setting, due to their high cost and the need for fundamental changes to the environmental architecture [18].

Within the group of wheelchair-based solutions, one can find lots of approaches, e.g., [19,20]. Interestingly, there is a clear subgroup in these robotic systems that is focused on the integration of robotic arms into the wheelchair [21,22,23]. In [24] we even can find a commercialized solution able to help with some ADLs. However, these solutions are specific for users with important restrictions in mobility, and their cost is far from being affordable for the general public.

Mobile platforms, the third group, have a dual purpose: service and companionship. Some examples of these mobile platforms can be found in [25,26,27,28,29]. They mainly focus on elderly people, to enable health monitoring. The approach we present in this work belongs to this third group. In contrast to [27,29], we do not need any expensive commercial platform to be adapted. We introduce a novel mobile low-cost robotic platform, which has been specifically designed to assist children with ADLs, by embedding an AI based module for online action monitoring.

With respect to the integration of action recognition solutions in ARDs, different studies revealed that this capability is fundamental for the interactions with the users [30,31,32,33,34,35,36,37,38,39]. Most of the approaches need a set of sensors to be installed in the home of the user, e.g., [30,31,32]. One can also find works where the activity recognition is performed with the use of body sensors, e.g., [33,34]. However, these solutions usually have low acceptance by the users.

Finally, some works offer a real-time action recognition capability using the cameras installed directly within the robotic platforms [35,36,37,38].

In [35] the authors introduced the PHAROS robot. This platform records subjects while exercising. Then, it produces data that are fed to a system to recognize the type of exercise being performed, and generates a sequence of exercises encapsulated in each user’s daily schedule. In [36], Zlatintsi et al. presented a platform to aid elderly people with bathing tasks. In [37], the authors described how a deep reinforcement learning approach can be useful to improve a robotic drinking assistant. The action recognition capability can be also used as the main human robot interface. See, for instance [38], where gestures were used to help people with multiple sclerosis. These works demonstrate the importance of integrating real-time action detection solutions to go a step further in robotic solutions for aiding with ADLs.

Previous works show that there are still some user needs that have not been properly covered. First, an ARD should be as simple as possible. Ideally, we should avoid having to install complex sensor networks at home or over the user’s body. Moreover, there is no doubt that within the context of ARDs, cost-effectiveness has to be taken into account, due to the fact that the final provision of the ARD to the user, usually, results in an expense payed by families or health services. In this sense, we provide a low-cost mobile platform that simply interacts with the user using a camera. There are other works that focus on the development of low-cost robotic platforms that provide real-time human action recognition, e.g., [39]. However, the differences from our approach are noteworthy: (a) while in [39] a Turtlebot commercial platform was used, we propose our own robotic design; and (b), ours is an ARD, which has been specifically designed to assist children with NDDs, with two applications based on action recognition monitoring.

Overall, our robot incorporates state-of-the-art online action recognition and navigation capabilities to interact with and monitor the users with NDDs. Our developed applications assist the users in order to improve their independence in ADLs. Although we focus in this study on children and youth, our technology could also be considered for elderly and disabled adults, who may also benefit from ARDs for improving functionality and autonomy in ADLs [4,11,18,40].

## 3. The Low-Cost, Assistive AI Robotic Platform

### 3.1. Description of the Hardware

**Figure 1:**
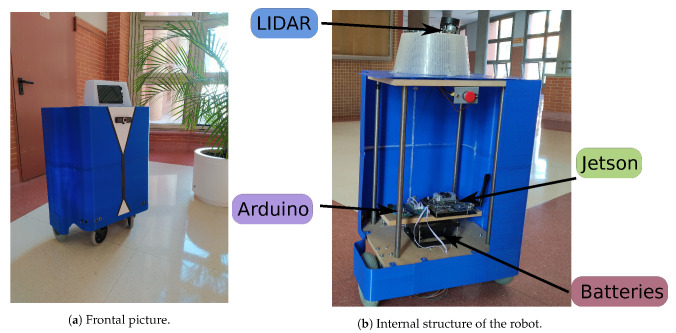
Pictures of our low-cost robotic platform. We show both a frontal picture and the internal structure, where it is possible to observe all the electronic and mechanical components of the platform.

One of the main contributions of our work is the construction of a new low-cost, assistive robotic platform, which can be seen in Figure 1.

It is a differential wheeled robot, equipped with two motors and their corresponding encoders, which are all controlled with an open-source Arduino board. All mechanical and electrical design has been performed by us. The internal structure is constructed of wood and metal. Additionally, the outer shell, imitating a person wearing a tuxedo, was made entirely by 3D printing. The platform is powered by two batteries, and it includes an electronic driver interface to allow easy interconnection of the different parts of the system and all the power management. All the electrical system can be powered with 24 or 12 Volts. For our implementation, we have opted to use 12 Volts to reduce the maximum speed that the motors can provide. Powered wheels are 190 mm in diameter and 590 mm is the axis distance. The complete platform measures approximately 800 mm, slightly higher than a table.

As for the sensors, the platform has the following: 1 LIDAR, a touch screen and a frontal camera. In order to integrate into the mobile robot all the high-level processing that cannot be embedded into the Arduino, the platform has a Jetson TX2 board from NVIDIA. This is a 7.5 watt computer on a single module that provides a GPU with 8 GB of memory. We have integrated the following systems into the NVIDIA Jetson board: (a) navigation; (b) visual perception; and (c) online action detection. In addition, it is on the Jetson where all the high-level applications that interact with the end users of the platform are executed.

Table 1 offers an estimation of the cost to build our platform, which is around 800 €. All details for building and replicating our low-cost platform will be publicly released. Our goal is that this low-cost technology could be replicated and adapted to all those who need it.

### 3.2. Software Architecture

**Figure 2:**
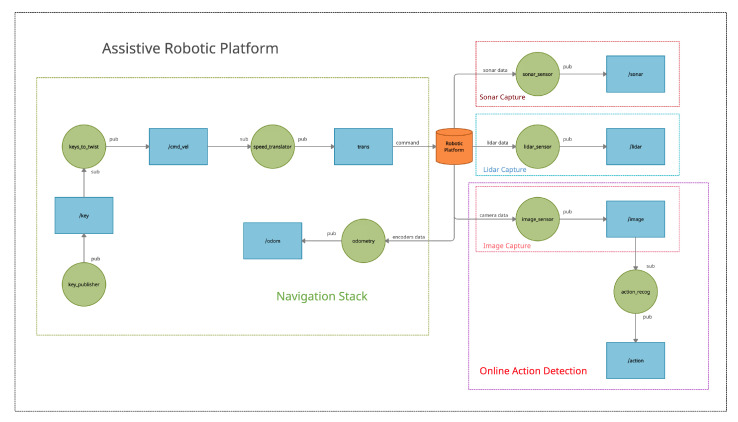
Robotic Operating System (ROS)-based complete software architecture.

All the software for the robotic platform has been fully developed under the Robotic Operating System (ROS) [41], which is an open source robotics middleware suite. ROS enables us to scan all the sensors on the robotic platform (cameras, motor encoders, LIDAR, Arduino sensors, etc.) at a given frequency, and to collect all the data to be processed. The key concept of ROS as a robotic operating system is to run a large number of nodes in parallel that can synchronously (or asynchronously) exchange information. Figure 2 shows the whole ROS-based architecture.

We have developed specific ROS nodes to directly communicate with our Arduino board, to take information from motor encoders and to control speed and movement of the wheels. These nodes form part of the ROS navigation stack, which allows us to initialize the platform localization system, and to send goals to the robot using a pre-built map of the environment. The architecture also integrates specific ROS nodes for reading both the LIDAR and the RGB camera. Additionally, finally, we also integrated our online action detection software into an ROS node. This way, we are able to publish action recognition messages into the ROS architecture, which are then used by the applications developed to attend kids with NDDs.

We used a pre-built map of the environment to allow the robot to navigate. Our map consisted of an image with the basic structure of the building in which the platform had to navigate. This image contained only black pixels where there were structural elements, such as walls and columns. Therefore, the map was an scaled version of the plan view of the building where the platform had to operate. The only information supplied to the robot was the scale to relate the distance between pixels and the distance in the real world. Once a target is set on such a map, which is the point where the final user is located, the platform is able to reach it to start monitoring the activity with the online action detection software.

The proposed software architecture is versatile. It is the core that enables us to deploy several applications for the care of children with NDDs based on the monitoring of their activity.

### 3.3. Online Action Detection

To implement any application that allows the monitoring of human activity by our robot, it is essential to integrate an online action detection (OAD) solution into it.

Traditionally, the task of action detection in videos has been addressed mainly from an offline perspective, e.g., [42,43,44,45,46,47,48,49,50,51]. These offline models basically assume that they dispose of the entire video in which the action takes place in order to perform the action detection. However, this offline scenario does not seem feasible for an application such as the one we propose to address in this project.

Think of our robotic platform. It has to interact with people in a practical way, being able to recognize the actions they make as soon as they are performed. It is therefore a matter of anticipating when the actions start and also of quickly detecting when they end. All previous offline approaches would not be able to work in the application scenario described. The reason is simple: they would detect action situations far later they their occurrence.

Hence, in this work we need to focus on the online perspective to address the action detection problem. OAD was introduced by De Geest et al., [52]. The goal of an OAD model is to predict actions as soon as they occur. There have been few works addressing this novel online setting, e.g., [43,52,53,54,55].

**Figure 3:**
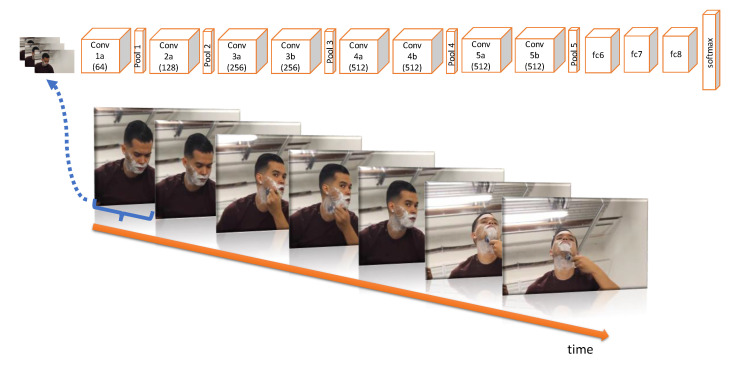
This figure shows the deep learning model OAD-3D-CNN, which is used for the monitoring of the daily-life activities of the users. Our model has 8 3D convolutions, 5 max-pooling layers and 3 fully connected layers, followed by a softmax output layer. All 3D convolution kernels are 3×3×3 with stride = 1 in both spatial and temporal dimensions. Note that in each box we indicate the number of filters. The 3D pooling layers are denoted pool1 to pool5, where the pooling kernels are 2×2×2, except pool1, for which it is 1×2×2. The fully connected layers have 4096 output units.

For this project, we have decided to integrate into the robotic platform our own OAD model [55], named OAD-3D-CNN. Technically, we propose a 3D convolutional neural network (CNN) approach inspired by the deep learning architecture detailed in [56], due to its success shown on the UCF101 dataset [57] for action recognition. Figure 3 shows all the details of the deep learning architecture deployed in our robotic platform.

Note that we apply 3D convolutional blocks directly to the input video volume (using chunks of 16 frames). This step results in another volume which preserves the temporal information of the input signal. Moreover, as it is shown in Figure 3, our model is trained to directly identify the actions in each frame of a video, hence identifying the actions as soon as the occur. Although the authors of [56] propose to perform the classification of the actions with an SVM classifier over fc6 features of the CNN model, we directly use the output of the final softmax layer to classify input chunks. This way, our approach is able to produce action estimations at more than 5 frames per second using the Jetson TX2 board for a live video stream obtained with the frontal camera of the robot. The implementation of the OAD-3D-CNN model has been done using PyTorch [58].

## 4. Monitoring ADLs

The main objective of our research consists of providing a low-cost assistive robotic platform with an embedded AI-based online action recognition approach to aid children and youth with neurodevelopmental disorders. This assistance is provided by the monitoring of ADLs performed by users.

In this section we first detail the procedure we followed to train and to validate an OAD module so as to perform the monitoring of the ADLs of the users (Section 4.1). Finally, Section 4.2 describes the implemented applications and a further discussion of the impact the applications will have on users with NDDs.

### 4.1. Teaching an OAD Module with Daily Living Activities: Experimental Validation

In order to implement the described monitoring, it was necessary to train our AI action recognition model to identify the actions of interest—daily living actions. However, the fact is that there are many databases for action recognition (e.g., [57,59,60,61,62]), but most of them provide few actions that are valid for the described scenario.

**Figure 4:**
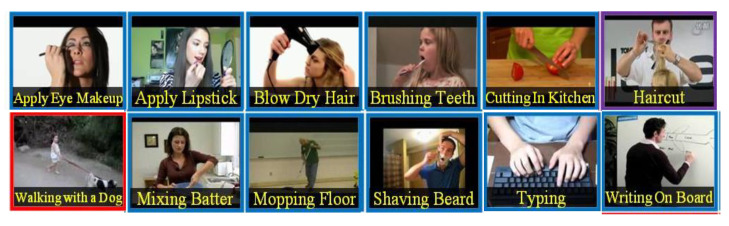
Visual examples of the 12 different action categories from the UCF-101 dataset used in our online action detection module.

For this research, we have opted to use the dataset UCF-101 [57]. It is a well-known action recognition dataset, which includes realistic action videos that were collected from YouTube. If provides 101 different action categories for 13,320 videos. For our project, we have made a selection from the 101 original categories provided with the dataset, choosing those of interest for monitoring kids with NDDs in our applications. In total, our model works with 12 different action categories. Table 2 shows the categories considered, and in Figure 4 we show some visual examples for them.

Technically, the OAD module in charge of the user monitoring can cast a prediction for one of the categories in Table 2 for every set of processed video frames. If none of them are detected, our OAD model produces a background category, i.e., no action of interest is taking place. All this information is dumped in a log file, where the output of the action recognition module is detailed for each time instant. This will be then used to deploy the monitoring applications.

We needed to train our Pytorch-based OAD-3D-CNN deep learning model on the UCF-101 dataset. For doing so, our network was fed with 16-frame length clips (and their corresponding action labels) randomly sampled from the training videos provided with UCF-101. Note that we used the official training + testing *Split 1* detailed in [57]. Weights from an initial training using Sports-1M dataset [62] were used to initialize the OAD-3D-CNN architecture, and SGD was configured as the optimizer.

To validate our model, we performed an experiment on the test set of *Split 1* in UCF-101. Each video in this test set was classified by evaluating the output of the OAD-3D-CNN model when it was fed with a random 16-frame clip sampled from the video. A 72.4% average clip accuracy was reported for *all* the 101 action categories in the dataset.

### 4.2. Assistance in ADLs with the Platform: Applications, Qualitative Results and Discussion

**Figure 5:**
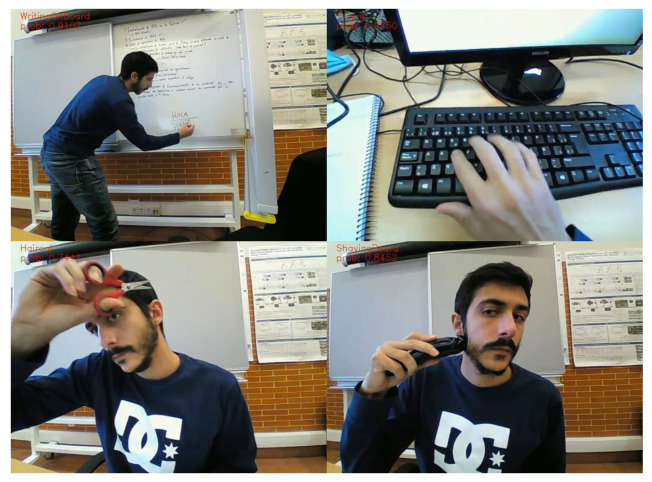
Qualitative results of the online action detection module. In this figure we show four images with the results of the action detection, which appear in the upper left margins, where we indicate the category recognized and the confidence of the OAD system for the prediction.

As we have previously described, our low-cost robotic platform can monitor the users in a known environment, for which a map is available. The application constructed uses the OAD module to cast an action label for every video frame. With the low-cost robotic platform proposed, embedding all the AI processing in the Jetson TX2 board, this can be done at 5 frames per second, a speed similar to previously reported results [63]. Figure 5 shows some qualitative results of the action detection module.

Finally, all the information generated by the OAD module is dumped into a log file, in which all the information related to a user is stored. These log files are then used to obtain the following statistics:

1. How much time has the user spent on certain tasks or actions?
2. What actions are carried out in certain time slots?
3. What actions are the most frequent?
4. How does the user sequence the different tasks or actions?

Being able to access this information is of great importance for health professionals who work with people with special needs, in terms of: selecting and measuring interventions; establishing treatment goals and outcomes.

Besides, the robotic platform also provides the user with real-time information regarding the action he/she is performing. This aspect makes it possible to create the following set of monitoring applications that we expect will have a special impact on improving environment participation in ADLs for users with neurodevelopmental disorders:

- Application 1—**Help for correct sequencing**. We implemented an application that monitors the sequencing of the activities. In other words, our platform will recognize in real time the actions that are being carried out, and will inform the user of what task should be the next one. E.g., if you are now shaving your beard, next step should be to inform you to start brushing your teeth. Further on, this help in sequencing actions could address more complex activities, such as “getting ready for school” or “going to the park with friends”. In this context, our robotic platform could indicate what to do first and how to do it, and then accompany the child in the transfers between one task to another, in order to work as a robot companion to facilitate independent mobility.
- Application 2—**Aid in action learning**. This application allows one to reinforce the learning of the correct performance of daily living activities. The robot simply monitors the action that is being performed, recognizing it. Once the action has been identified, the robot can show the users videos with people performing the same type of action, with the intention of reinforcing in them how to develop the action in an adequate way.

## 5. Conclusions

In this paper we have introduced a novel low-cost robotic platform which has been specially designed to improve the environment participation and function in daily living activities for children with neurodevelopmental disorders. Our robot integrates a sophisticated online action detection module which is able to monitor the actions performed by the users, in order to aid them with both the sequencing and learning of the daily living activities.

In this work we describe all the technical details that make possible the implementation of the described applications to assist kids with NDD. As an immediate future line of work, we would like to measure the impact that the use of the platform and the described applications has on end users with NDDs. For doing so, we have established some collaborations with different centers where we will be able to test the proposed technology with kids and youth.

Another important future line of work will consist in enriching the action recognition capabilities of the robot, in order to deal with more daily living action categories. Technically, we plan to include in the training dataset more videos containing actions of interest for our users. This video gathering step will be done by inspecting the categories provided by other databases for action recognition, or even by recording our own sequences.

## Figures and Tables

**Table 1:** A list with the prices of the main components needed to build our platform.

Item	Estimated Price
Motor and encoders	132 €
Arduino MEGA	12 €
Battery	24 €
Wheels	66 €
Structure & Components	100 €
Screen	40 €
Jetson TX2	320 €
Camera	20 €
LIDAR	90 €
Total	804 €

**Table 2:** A list detailing the subset of the action categories from the UCF-101 dataset that we have implemented in the online action detection (OAD) software module. We also report the number of videos available for training.

Category	UCF-101 Class Identifier	Number of Videos
Apply Eye Makeup	1	>130
Apply Lipstick	2	>100
Blow Dry Hair	13	>120
Brushing Teeth	20	>120
Cutting In Kitchen	25	>100
Haircut	28	>120
Mixing Batter	35	>130
Mopping Floor	55	>100
Shaving Beard	56	>150
Typing	95	>120
Walking With Dog	96	>120
Writing On Board	100	>150

## Data Availability

UCF-101 [57] dataset can be accessed at https://www.crcv.ucf.edu/data/UCF101.php, accessed on 9 April 2021.

## Abbreviations

The following abbreviations are used in this manuscript:

ADLs	Activities of Daily Living
ICF	International Classification of Functioning, Disability and Health
AT	Assistive Technology
NDD	Neurodevelopmental Disorder
ARD	Assistive Robotic Device
CNN	Convolutional Neural Network
OAD	Online Action Detection
ROS	Robot Operating System
LD	Linear dichroism
